# Identification of OsGGR2, a second geranylgeranyl reductase involved in α-tocopherol synthesis in rice

**DOI:** 10.1038/s41598-018-19527-3

**Published:** 2018-01-30

**Authors:** Eiichi Kimura, Takumi Abe, Kazumasa Murata, Toshiyuki Kimura, Yurika Otoki, Taiji Yoshida, Teruo Miyazawa, Kiyotaka Nakagawa

**Affiliations:** 1 National Agricultural Research Center for Tohoku Region, NARO, Morioka, Iwate, 020–0198 Japan; 2 Food and Biodynamic Chemistry Laboratory, Graduate School of Agricultural Science, Tohoku University, Sendai, Miyagi 980 – 0845 Japan; 3 Agricultural Research Institute, Toyama Prefectural Agricultural, Forestry and Fisheries Research Center, Toyama, Toyama, 939–8153 Japan; 4 Division of Food Function Research, Food Research Institute, NARO, Tsukuba, Ibaraki, 305–8642 Japan; 5 National Agricultural Research Center for Tohoku Region, NARO, Morioka, Iwate, 020–0198 Japan; 6 Food and Biotechnology Innovation Project, New Industry Creation Hatchery Center (NICHe), Tohoku University, Sendai, Miyagi 980–8579 Japan

## Abstract

Tocopherol (Toc) and tocotrienol (T3) are abundant in rice bran. Geranylgeranyl reductase (GGR) is an essential enzyme for Toc production that catalyzes the reduction of geranylgeranyl pyrophosphate and geranylgeranyl-chlorophyll. However, we found that a rice mutant line with inactivated *Os02g0744900* (*OsGGR1/LYL1/OsChl* *P*) gene produces Toc, suggesting that rice plants may carry another enzyme with GGR activity. Using an RNA-mediated interference technique, we demonstrated that the *Os01g0265000* (“*OsGGR2*”) gene product has GGR activity. This result supports the existence of two *GGR* genes (*OsGGR1* and *OsGGR2*) in rice, in contrast to *Arabidopsis thaliana* (thale cress) and cyanobacterium *Synechocystis* that each have only one *GGR* gene. We also produced rice callus with inactivated *OsGGR1* and *OsGGR2* that produced T3 but not Toc. Such rice callus could be used as a resource for production of pure T3 for nutraceutical applications.

## Introduction

Tocopherol (Toc) and tocotrienol (T3) are both forms of vitamin E, which was discovered more than 90 years ago when its absence in the diet was shown to promote sterility in mice^1^. Vitamin E is biosynthesized in the photosynthetic organs of plants and cyanobacteria and plays essential roles in both plant and animal physiology^2,3^. Many genes involved in vitamin E biosynthesis in plants have been identified, including *VTE* (*VITAMIN E*) 1^4,5^, *VTE2-1*^6,7^, *VTE2-2*^8,9^, *VTE3*^10^, *VTE4*^11^, *VTE5*^12^, *VTE6*^13^, *HGGT* (*homogentisic acid geranylgeranyl transferase*)^14^, and *PPH* (*pheophytinase*)^15^.

Geranylgeranyl reductase (GGR) plays crucial roles in both vitamin E and chlorophyll biosynthesis. GGR is thought to have two functions: reduction of geranylgeranyl pyrophosphate (GGPP) to phytyl pyrophosphate (PPP), and reduction of geranylgeranyl-chlorophyll to chlorophyll^16^. *Arabidopsis thaliana* encodes only one *GGR* gene at the gene locus At1g74470. The homologous gene to *Arabidopsis GGR* in rice (*Oryza sativa*) is *Os02g0744900* (*OsGGR1/LYL1/OsChl* *P*), which has a nucleic acid sequence that is 65% identical to that of *At1g74470*.

T3 is characteristically abundant in rice bran and is known to have greater antioxidant activity^17^, triglyceride-lowering effects^18^, and anti-angiogenesis activity^19^ compared to Toc. As such, we aim to develop new varieties of T3-rich rice to produce high-purity T3 without Toc. During this effort, we previously described rice cultivars that are rich in T3^20^, and used quantitative trait loci (QTL) analysis to identify five loci on rice chromosomes that contribute to T3 production^21^.

**Figure 1:**
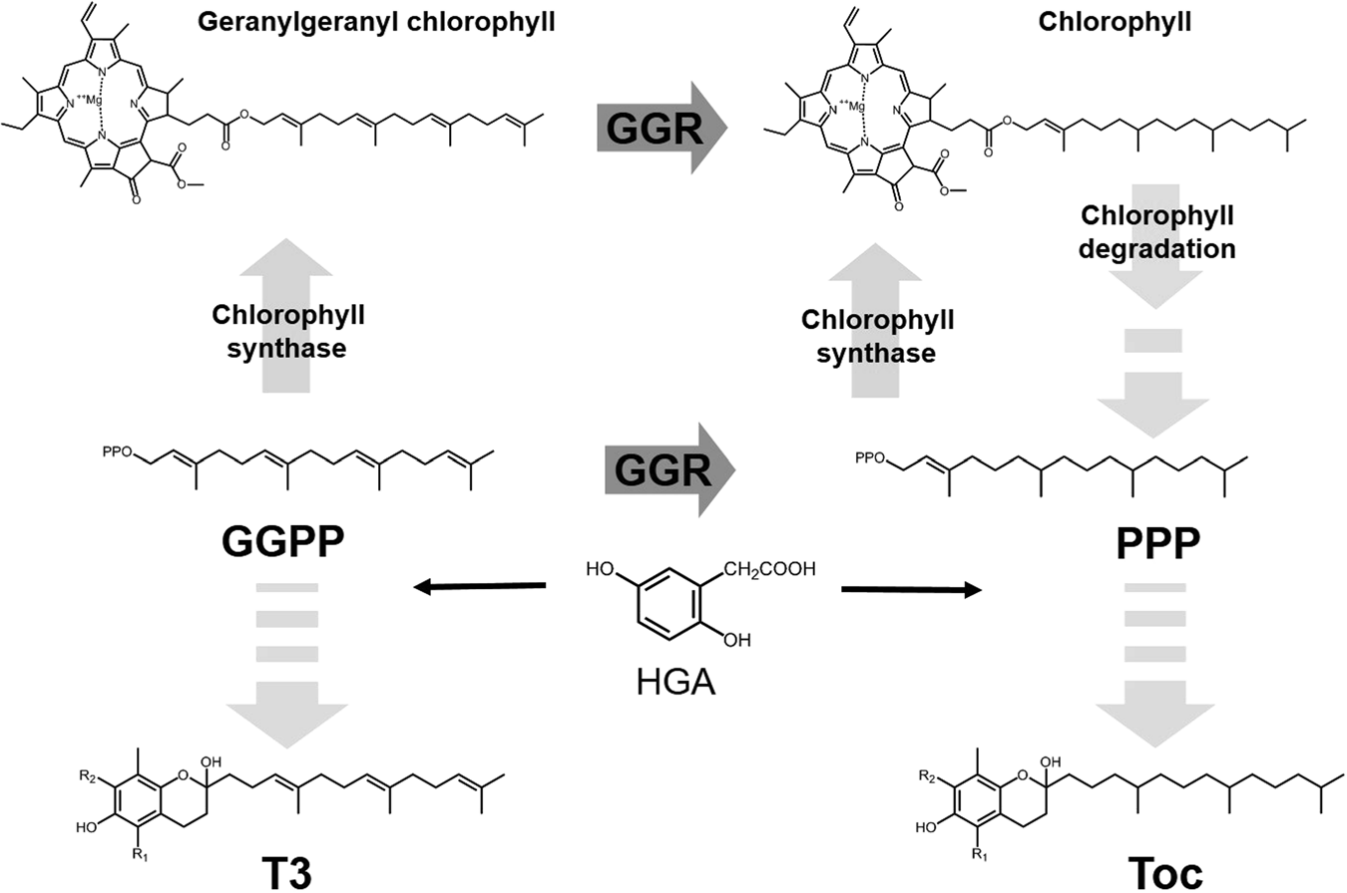
Schematic pathway of vitamin E biosynthesis in plants. Abbreviations: Toc, tocopherol; T3, tocotrienol; GGPP, geranylgeranyl pyrophosphate; PPP, phytyl pyrophosphate; HGA, homogentisic acid; HGGT, homogentisate geranylgeranyl transferase; GGR, geranylgeranyl reductase. These enzymes, chlorophyll synthase, and chlorophyll degradation related enzymes are involved in vitamin E biosynthesis in plants. R1and R2 represent methyl groups or hydrogen.

T3 is biosynthesized from GGPP and homogentisic acid (HGA), whereas Toc is biosynthesized from PPP and HGA. PPP is generated from GGPP via the chlorophyll degradation pathway or direct reduction of GGPP by GGR catalytic activity (Fig. 1). We predicted that if *GGR* is inactivated, GGPP levels would increase, accompanied by an increase in T3 and absence of Toc synthesis. During the analysis of rice with an inactive *GGR* mutation, we found evidence for the existence of a second gene involved in *GGR* synthesis in addition to the Arabidopsis *GGR* orthologue *OsGGR1*.

In this study, we analyzed the *GGR* gene in rice and showed that rice has two *GGR* genes (*OsGGR1* and *Os01g0265000* (“*OsGGR2*”)). We also showed that when both genes are inactivated in rice callus, Toc biosynthesis is eventually inhibited.

## Results

### Phenotypes of OsGGR1 Tos17 mutant rice

**Figure 2:**
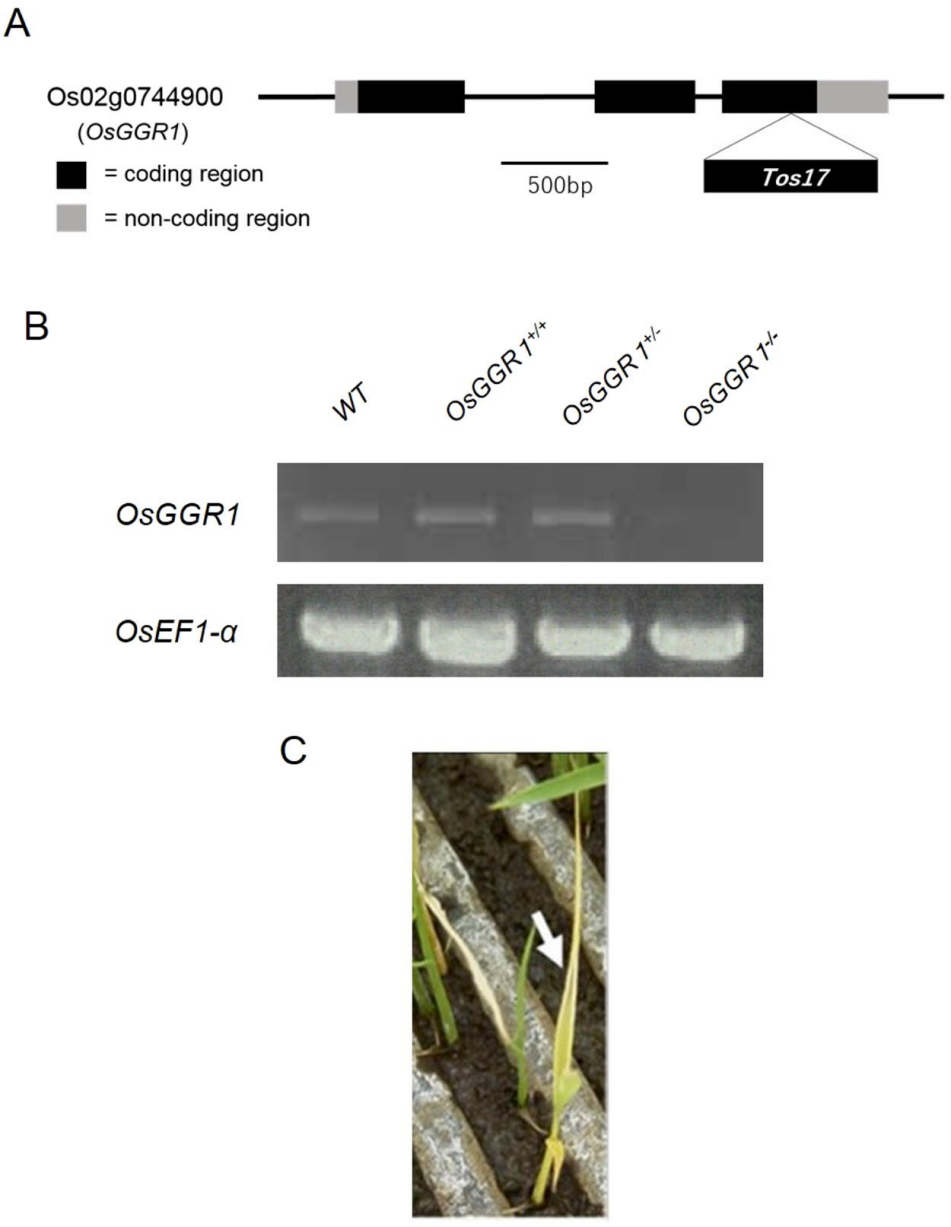
Analysis of *Tos17* mutant rice line NE1041 with inactive Os02g0744900 (OsGGR1). (**A**) Exon-intron gene structure of the *OsGGR1 Tos17* mutant line (NE1041). Coding region and *Tos17*-inserted region are in black and non-coding region is in gray. (**B**) Transcript expression level of *OsGGR1* in *OsGGR1 Tos17* homozygous and heterozygous mutant. The full-length blots were presented in Supplementary Figure S1. (**C**) Pale phenotype of *OsGGR1 Tos17* homozygous mutant rice.

*Arabidopsis thaliana* has one gene encoding *GGR* (*AtGGR*). In rice, *OsGGR1* (*Os02g0744900*) is an *AtGGR* (*At1g74470*) orthologue. Using the rice line with a retrotransposon *Tos17* insertion mutant^22^ of *OsGGR1* (NE1041) (Rice Genome Resource Center, National Agriculture and Food Research Organization [NARO], Tsukuba, Japan) (Fig. 2A), we divided the genotypes of *OsGGR1 Tos17* mutant rice seedlings into three groups: *OsGGR1*^+/+^ (wild-type [WT] homozygous genotype), *OsGGR1*^+/−^ (heterozygous genotype), and *OsGGR1*^−/−^ (mutant homozygous genotype), and confirmed that the *OsGGR1*^−/−^ homozygous mutant did not express *OsGGR1* mRNA (Fig. 2B). *OsGGR1*^−/−^ rice seedlings also displayed an incomplete albino phenotype under direct sunlight (Fig. 2C).

### Quantitative analysis of Toc and T3 content in leaves and callus of OsGGR1 mutant rice

**Figure 3:**
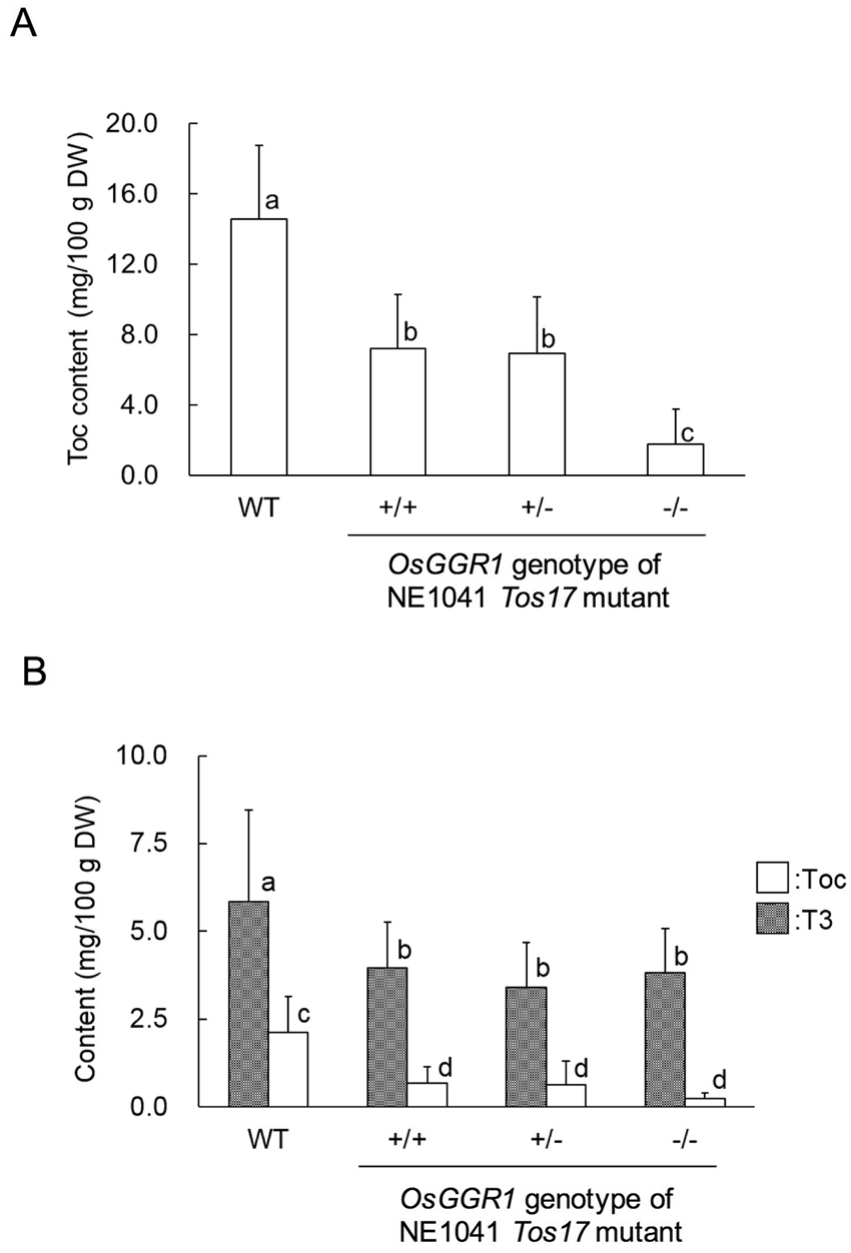
Toc and T3 content in rice leaf and callus from WT and *OsGGR1 Tos17* mutant line NE1041. (**A**) Toc content in rice leaf of WT and three genotypes of *OsGGR1 Tos17* mutant line NE1041. Values are means ± SD; WT, *n* = 6; +/+ , *n* = 19; + /−, *n* = 46; −/−, *n* = 30. (**B**) Toc and T3 content in rice callus of WT and three genotypes of NE1041. Values represent total concentrations of individual Toc isomers or T3 isomers. Values are means ± SD; WT, *n* = 7; +/+, *n* = 8; +/−, *n* = 8; −/−, *n* = 8. Labeled means without a common letter differ, *p* < 0.05. (Kruskal-Wallis H-test followed by the Student-Newman-Keuls test). WT = wild-type; DW = dry weight.

We analyzed the foliar vitamin E content of the three genotypes of the *Tos17* mutant and WT. Although the Toc content in the OsGGR1^−/−^ mutant was significantly decreased compared with the other *Tos17* mutants (*OsGGR1*^+/+^ and *OsGGR1*^+/-^) as well as WT, we confirmed the presence of substantial amounts of Toc in the OsGGR1^−/−^ mutant (Fig. 3A). T3 was not detected in the leaf samples. We also analyzed callus generated from the *OsGGR1 Tos17* mutants and WT plants. Similar to the rice leaves, Toc in callus was present in the *Tos17* mutants and WT (Fig. 3B). T3 was detected in the callus samples, although the amount of T3 in the *Tos17* mutants was lower than that of WT.

### Expression analysis of OsGGR2 mRNA

**Figure 4:**
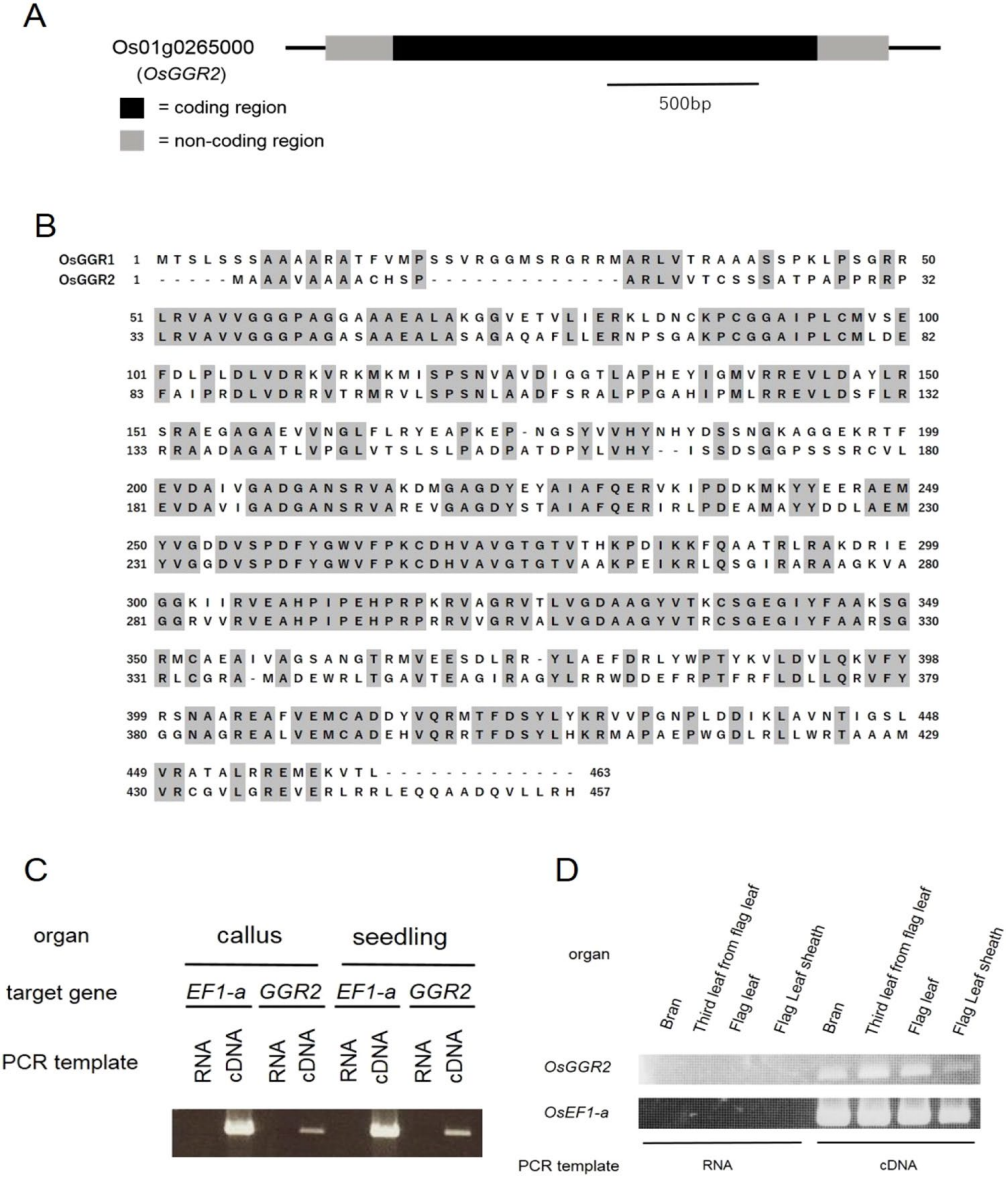
Gene expression analysis of *Os01g0265000* (*OsGGR2*). (**A**) Gene structure of *OsGGR2*. The black and gray portions represent the coding region and non-coding region, respectively. (**B**) Amino acid sequence alignment of OsGGR1 and OsGGR2. (**C**) RT-PCR analysis of *OsGGR2* gene expression in rice callus and seedlings. The positive control gene was *OsEF1-α* and a negative control was performed using DNase I-treated RNA template. The full-length blots were presented in Supplementary Figure S2. (**D**) RT-PCR analysis of *OsGGR2* gene expression in mature rice organs. The full-length blots were presented in Supplementary Figure S3.

A Basic Local Alignment Search Tool (BLAST) search analysis revealed that rice has another gene that is similar to *OsGGR1*. We designated this *OsGGR1* homologue *Os01g0265000* as “*OsGGR2*”. We next analyzed *OsGGR2* mRNA expression because this gene is not registered in the full-length cDNA library database KOME (Knowledge-based Oryza Molecular Biological Encyclopedia) of NARO^23^ (database now unavailable). We performed reverse transcriptase (RT)-PCR using the predicted sequences of the 5′ and 3′ non-coding regions as primers (Table S1). *OsGGR2* was indeed expressed at the mRNA level in the callus and leaves of seedlings (Fig. 4C). The 1,374 bp nucleotide sequence of the cloned *OsGGR2* mRNA coding region is GC-rich (76%) and lacks introns (Fig. 4A). Relative to *OsGGR1*, the *OsGGR2* sequence has 64% and 53% similarity at the nucleic acid and amino acid level, respectively. Amino acid sequence alignment of OsGGR1 and OsGGR2 is presented in Fig. 4B. Further *OsGGR2* expression pattern analysis in the grain filling stage showed that *OsGGR2* is expressed in bran, the flag leaf, the third leaf from the flag leaf, and the flag leaf sheath (Fig. 4D).

### Functional analysis of OsGGR2 in the Toc biosynthetic pathway

**Figure 5:**
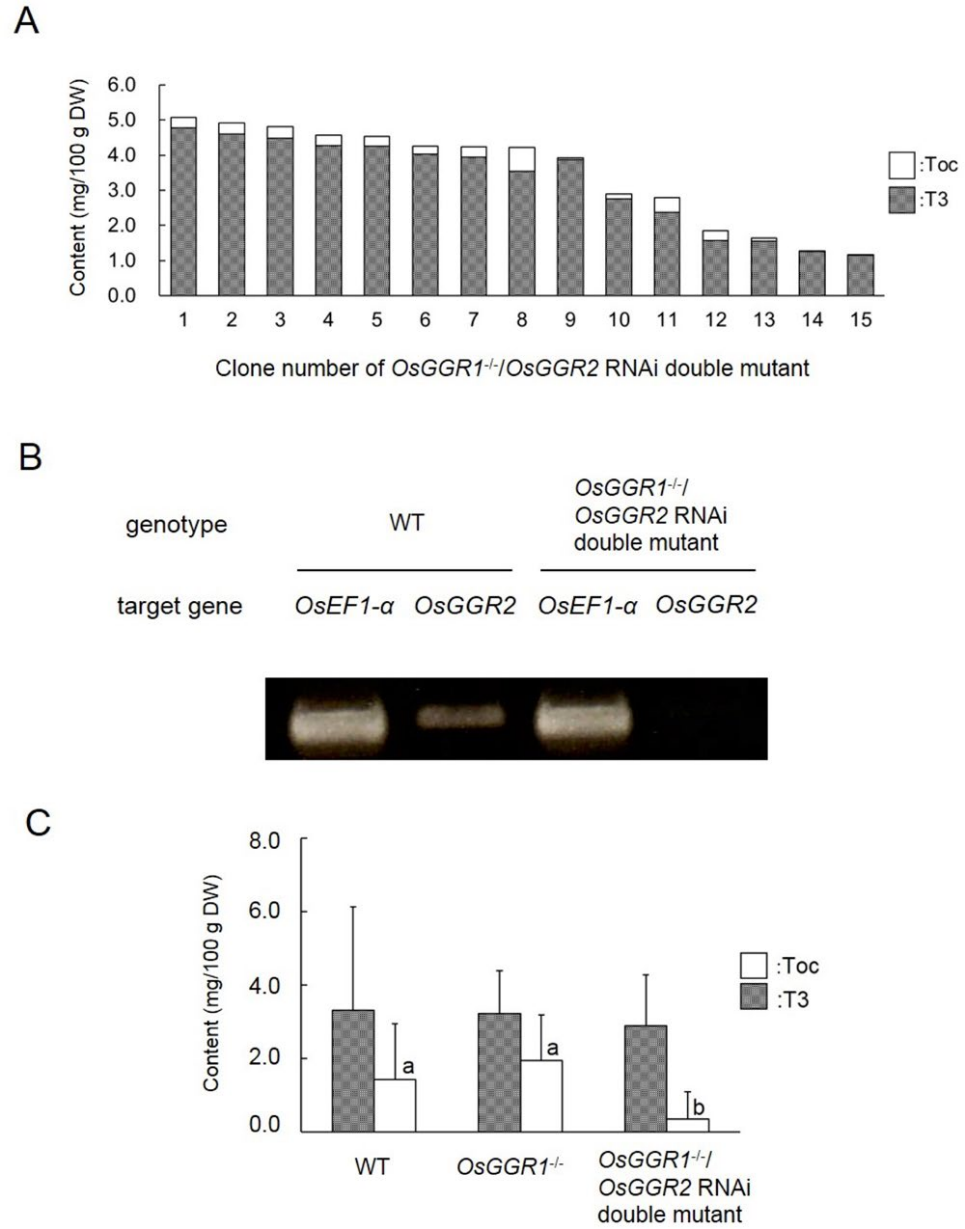
Functional analysis of OsGGR2 activity in rice vitamin E biosynthesis. (**A**) Toc and T3 content in *OsGGR1*^−/−^/*OsGGR2* RNAi double mutant rice callus. (**B**) *OsGGR2* gene expression analysis of WT and *OsGGR1*^−/−^/*OsGGR2* RNAi double mutant callus. The full-length blots were presented in Supplementary Figure S4. (**C**) Toc and T3 content of WT, *OsGGR1*^−/−^, and *OsGGR1*^−/−^/*OsGGR2* RNAi double mutant callus. Black and white bars indicate Toc and T3 content, respectively, in rice callus. Values represent total concentrations of individual Toc isomers or T3 isomers. Values are means ± SD; WT, *n* = 46; *OsGGR1*^−/−^, *n* = 35; *OsGGR1*^−/−^/*OsGGR2* RNAi, *n* = 28. Labeled means without a common letter differ, *p* < 0.05. (Kruskal-Wallis H-test followed by the Student-Newman-Keuls test). *OsGGR1*^−/−^ = *OsGGR1* inactive *Tos17* mutant. *OsGGR1*^−/−^/*OsGGR2* RNAi = *OsGGR1*^−/−^ and *OsGGR2* RNAi double mutant. WT = wild type. DW = dry weight.

Because the *OsGGR2* mutant was not included in the *Tos17* mutant panel, we reduced the expression level of endogenous *OsGGR2* in *OsGGR1*^−/−^ genotype *Tos17* mutant rice callus using an RNA-mediated interference (RNAi) technique to produce an *OsGGR1*^−/−^*/OsGGR2* RNAi double mutant. We then analyzed the vitamin E content of callus formed by the double mutant to assess *OsGGR2* involvement in vitamin E biosynthesis (Fig. 5A). Results for RT-PCR analysis of *OsGGR2* gene expression by a representative double mutant callus (clone No. 9) and average vitamin E content of WT callus, *OsGGR1*^−/−^ callus, and *OsGGR1*^−/−^/*OsGGR2* RNAi double mutant callus are shown in Fig. 5B and C. The Toc content of the double mutant callus was drastically reduced compared with WT and the *OsGGR1*^−/−^ callus. These results indicate that the *OsGGR2* gene product has GGR activity and synthesizes Toc in rice plant cells.

## Discussion

The vitamin E synthesis pathway (Fig. 1) has been elucidated mainly by studies using *Arabidopsis thaliana* and the *Synechocystis* mutant^4–13,15^. GGR was first identified in *Arabidopsis thaliana*^16^ as an essential enzyme in the biosynthesis of Toc and chlorophyll^24,25^. GGR reduces GGPP to PPP, and also reduce geranylgeranyl-chlorophyll to chlorophyll. In addition to the direct reduction of GGPP to PPP by GGR, hydrolytic cleavage of the chlorophyll phytyl side chain produces phytol, which is then phosphorylated to form PPP. Toc is biosynthesized from PPP and HGA by the catalytic action of VTE2–1, 2, whereas T3 is biosynthesized from GGPP and HGA by the catalytic action of HGGT.

In this study, we prepared *OsGGR1 Tos17* mutant rice samples. Since *GGR* is also necessary for chlorophyll production (Fig. 1), the phenotype of *OsGGR1*^−/−^ rice is incomplete albino (Fig. 2C), indicating that *OsGGR1* is inactivated in *OsGGR1*^−/−^ genotype rice. Moreover, *OsGGR1*^−/−^ *Tos17* mutant rice plants are sterile. Incidentally, *OsGGR1/LYL1/OsChl P* mutants isolated via ethylmethanesulfonate (EMS) mutagenesis or ^60^Co irradiation are fertile^26,27^. Mutations in the fertile mutants would thus be expected to have moderate effects.

Only one *GGR* gene is present in *Arabidopsis thaliana*, *Nicotiana tabacum*, and *Synechocystis*, which is consistent with the observation that a cyanobacterium mutant carrying inactivated GGR cannot grow photoautotrophically or produce Toc^28^. In this study, the *OsGGR1*^−/−^ *Tos17* mutant was also unable to grow under photoautotrophic conditions, but the mutant did produce substantial amounts of Toc (Fig. 3A**)**. Likewise, callus on *OsGGR1*^−/−^ plants contained Toc (Fig. 3B). Considering these findings (Fig. 3) and the biosynthesis pathway of vitamin E (Fig. 1), this result suggested that rice plants may carry another enzyme that has GGR activity. On the other hand, T3 was present in callus, but not in leaves (Fig. 3). This outcome is likely due to a lack of HGGT expression in rice leaves^29^.

We confirmed the existence of an *OsGGR1* homologue in the rice genome using a BLAST search and designated this gene as *OsGGR2* (*Os01g0265000*). We evaluated *OsGGR2* expression by RT-PCR because *OsGGR2* is not registered in the full-length KOME cDNA clone database (temporarily unavailable). The *OsGGR2* gene is expressed in several rice organs, including leaf and bran. The existence of both *OsGGR1* and *OsGGR2* and their preservation throughout evolutionary history suggests that the functions of these two genes are not redundant and cannot substitute for one another. One possibility for the presence of two rather than one gene is that OsGGR1 and OsGGR2 may be distributed among different cell compartments and work individually by our speculation.

To determine OsGGR2 function in rice cells, we generated *OsGGR1* and *OsGGR2* double mutant callus tissue by suppressing *OsGGR2* gene expression in *OsGGR1*^−/−^ genotype rice callus using RNAi. Toc content was drastically decreased in the double mutant callus compared with the *OsGGR1*^−/−^ single mutant, but did not reach zero (Fig. 5A,C). This residual production is likely because RNAi cannot completely abolish target transcripts. Moreover, gene expression inhibition efficiency is influenced by the rice genomic locus into which transfer-DNA (T-DNA) is integrated. These results further support the finding that rice has two active forms of GGR, OsGGR1 and OsGGR2.

According to the RiceXpro database^30^, *OsGGR2* expression is relatively stronger during the early embryo stage, suggesting that this gene might play an important role in early plant development. Meanwhile, *OsGGR1* is expressed strongly in leaves, which is consistent with the pale phenotype seen for the *OsGGR1 Tos17* mutant (Fig. 2C). As described above and as shown in Fig. 1, there are two pathways of PPP synthesis, although we did not investigate the extent to which OsGGR2 can contribute to Toc biosynthesis in the two PPP production pathways together with OsGGR1. Recently, Vom Dorp *et al*.^13^ reported on VTE6, which exhibits phytyl-phosphate kinase activity when PPP is produced in the chlorophyll degradation pathway. According to this report, in *Arabidopsis thaliana*, PPP production in Toc synthesis occurs mainly through the chlorophyll degradation pathway and not by direct reduction of GGPP. We are currently examining our gene silencing mutant of *OsGGR2* to further examine the functional differences between OsGGR1 and OsGGR2 in the two Toc biosynthesis pathways. In addition, enzymatic activity of OsGGR2, detailed comparison of gene expression level of *OsGGR1* and *OsGGR2*, and relationship between message level of *OsGGR2* and amount of Toc and T3 will be clarified in our future work.

Unlike Toc, T3 has potent anticancer activity by inhibiting angiogenesis^31^. T3 has thus attracted attention as a preventative and curative agent for diseases, as more than 50 diseases are associated with abnormal angiogenesis, including cancer, age-related macular degeneration and rheumatic diseases. The anticancer activity of T3 may be reduced by Toc through inhibition of its uptake^32^. Owing to their similar molecular structures, separating and purifying T3 from rice bran containing Toc is expensive and cumbersome. The production of Toc-free T3 produced from rice callus (Fig. 5A) would bypass these difficulties, and might be useful to generate pharmaceuticals aimed at suppressing angiogenesis. By generating callus with inactivation of both OsGGR1 and OsGGR2 activity, we showed that rice plant materials contained T3 but not Toc. This approach may provide a new pathway for the purification of T3 without Toc.

## Methods

### Genotype classification of rice Tos17 mutant of OsGGR1

Genomic DNA was isolated by ethanol precipitation of crushed rice leaves suspended in DNA extraction buffer (200 mmol Tris HCl pH 7.5, 250 mmol NaCl, 25 mmol EDTA). The DNA was subjected to a polymerase chain reaction (PCR) performed with appropriate primers (Hokkaido System Science, Inc. Sapporo, Japan) (Table S1).

### Quantitative analysis of vitamin E content

Vitamin E was extracted from rice samples with 2-propanol, and the extract was subjected to liquid chromatography with tandem mass spectrometry (LC-MS/MS) as described previously^33^. Separation was performed at 40 °C using a silica column (ZORBAX Rx-SIL, 4.6 × 250 mm; Agilent, Palo Alto, CA, USA). A mixture of hexane/1,4-dioxane/2-propanol (100:4:0.5) was used as the mobile phase at a flow rate of 1.0 mL/min. Toc and T3 were detected in atmospheric pressure chemical ionization mode (APCI). MS/MS parameters were optimized with Toc and T3 standards in APCI mode (positive). Toc and T3 were detected using multiple reaction monitoring as follows: α-Toc, *m/z* 431.3 > *m/z* 165.1; β-Toc, *m/z* 417.3 > *m/z* 151.3; γ-Toc, *m/z* 417.3 > *m/z* 151.0; δ-Toc, *m/z* 403.3 > *m/z*137.0; α-Toc-3, *m/z* 425.3 > *m/z* 165.1; β-Toc-3, *m/z* 411.3 > *m/z* 151.1; γ-Toc-3, *m/z* 411.3 > *m/z* 151.2; δ-Toc-3, *m/z* 397.2 > *m/z* 137.0. Toc and T3 concentrations in the rice samples were calculated using calibration curves for standard Toc and T3 concentrations.

### RT-PCR analysis of rice GGR expression

Total RNA was extracted with an RNeasy Plant Mini Kit^®^ (Qiagen, Hilden, Germany), followed by genomic DNA digestion with DNase I (TaKaRa, Shiga, Japan) at 37 °C for 30 min. The resulting total RNA was again purified with an RNeasy Plant Mini Kit to remove any remaining genomic DNA and DNase I. cDNA was synthesized from the total RNA using a QuantiTect^®^ reverse transcription kit (Qiagen) and subjected to PCR performed with appropriate primers (Table S1). We selected *OsEF1-α* as a positive control gene and used a DNase I-treated RNA template as a negative control.

### Transformation of rice callus

Transformation of rice callus was performed with *Agrobacterium* strain EHA 101 containing the gene silencing plasmid pANDA35HK. A partial sequence containing the 5′-noncoding 170 bp and 5′-coding 251 bp region of the *OsGGR2* gene was inserted into pANDA35HK, which was a generous gift from the late Dr. Shimamoto and Dr. Miki (former affiliation: Nara Institute of Science and Technology) as reported previously^34^. *Agrobacterium*-mediated transformation of rice callus was performed according to the method described by Toki *et al*.^35^.

### Statistical analysis

The data, expressed as mean ± SD, were subjected to the Kruskal-Wallis H-test followed by the Student-Newman-Keuls test. Statistical calculation was carried out using ystat 2000, an Excel statistical program file (IgakuTosho Shuppan, Tokyo, Japan). Differences with P < 0.05 were considered significant.

## Electronic supplementary material

Supplemental data
