# Supplementary material for: Identification of OsGGR2, a second geranylgeranyl reductase involved in α-tocopherol synthesis in rice
Source: Sci Rep. 2018 Jan 30;8:1870. doi: 10.1038/s41598-018-19527-3 (PMC5789843; doi:10.1038/s41598-018-19527-3)
Supplement: Supplementary file 1 — Supplemental data [file 41598_2018_19527_MOESM1_ESM.docx]

**SUPPLEMENTARY INFORMATION**

**Identification of OsGGR2, a second geranylgeranyl reductase involved in α-tocopherol synthesis in rice**

Eiichi Kimura^a^*, Takumi Abe^b^*, Kazumasa Murata^c^, Toshiyuki Kimura^d^, Yurika Otoki^b^, Taiji Yoshida^e^, Teruo Miyazawa^f^, Kiyotaka Nakagawa^b^**

^a^ National Agricultural Research Center for Tohoku Region, NARO, Morioka, Iwate 020-0198, Japan

^b^ Food and Biodynamic Chemistry Laboratory, Graduate School of Agricultural Science, Tohoku University, Sendai, Miyagi 980-0845, Japan

^c^ Agricultural Research Institute, Toyama Prefectural Agricultural, Forestry and Fisheries Research Center, Toyama, Toyama 939-8153, Japan

^d^ Division of Food Function Research, Food Research Institute, NARO, Tsukuba, Ibaraki 305-8642, Japan

^e^ National Agricultural Research Center for Tohoku Region, NARO, Morioka, Iwate 020-0198, Japan

^f^ Food and Biotechnology Innovation Project, New Industry Creation Hatchery Center (NICHe), Tohoku University, Sendai, Miyagi 980-8579, Japan

*equal contribution.

**Corresponding to: Dr Kiyotaka Nakagawa, E-mail: nkgw@m.tohoku.ac.jp.

**Supplementary Figures and Legends**

**
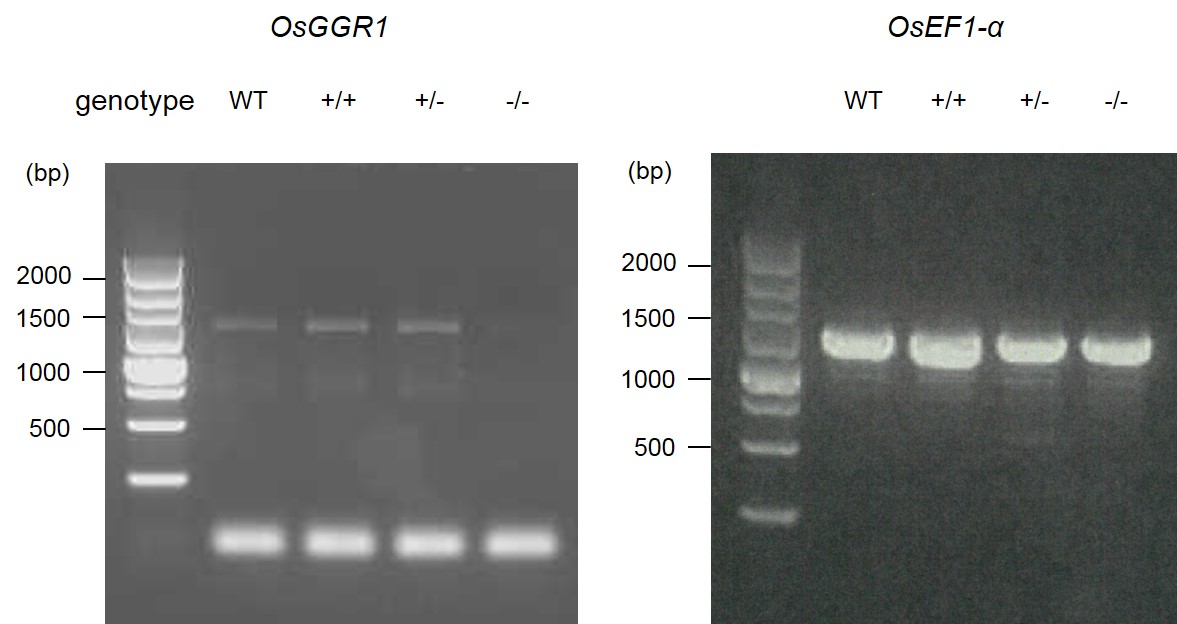
**

**Supplementary Figure S1. Full-length Gel from the article of Fig. 2B.**


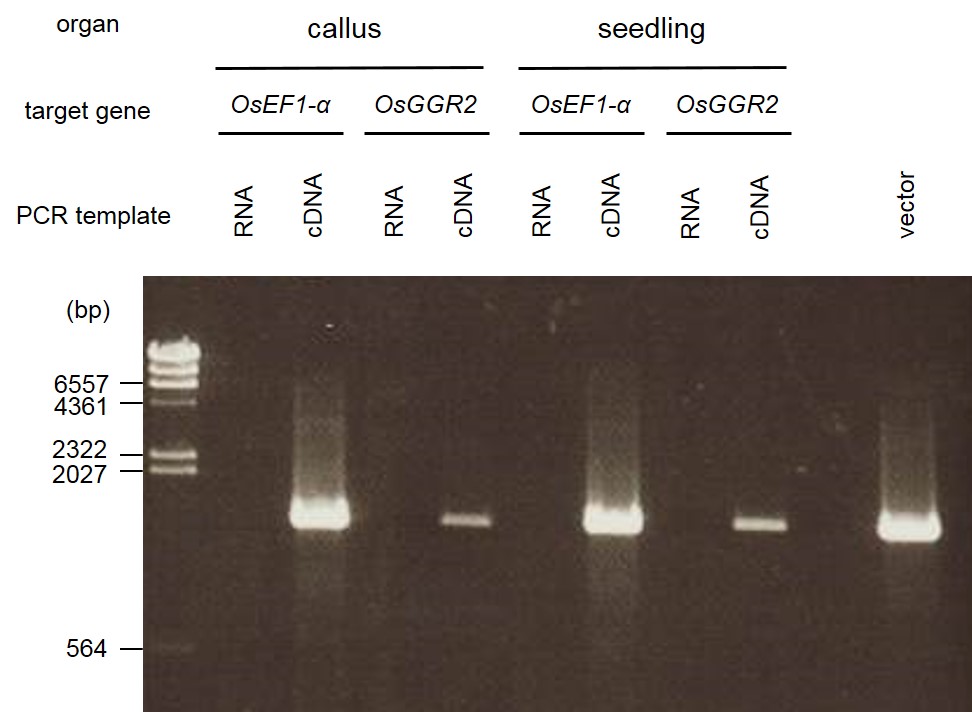


**Supplementary Figure S2. Full-length Gel from the article of Fig. 4C.** Vector is positive control of PCR template containing *OsGGR2* gene sequence.

**
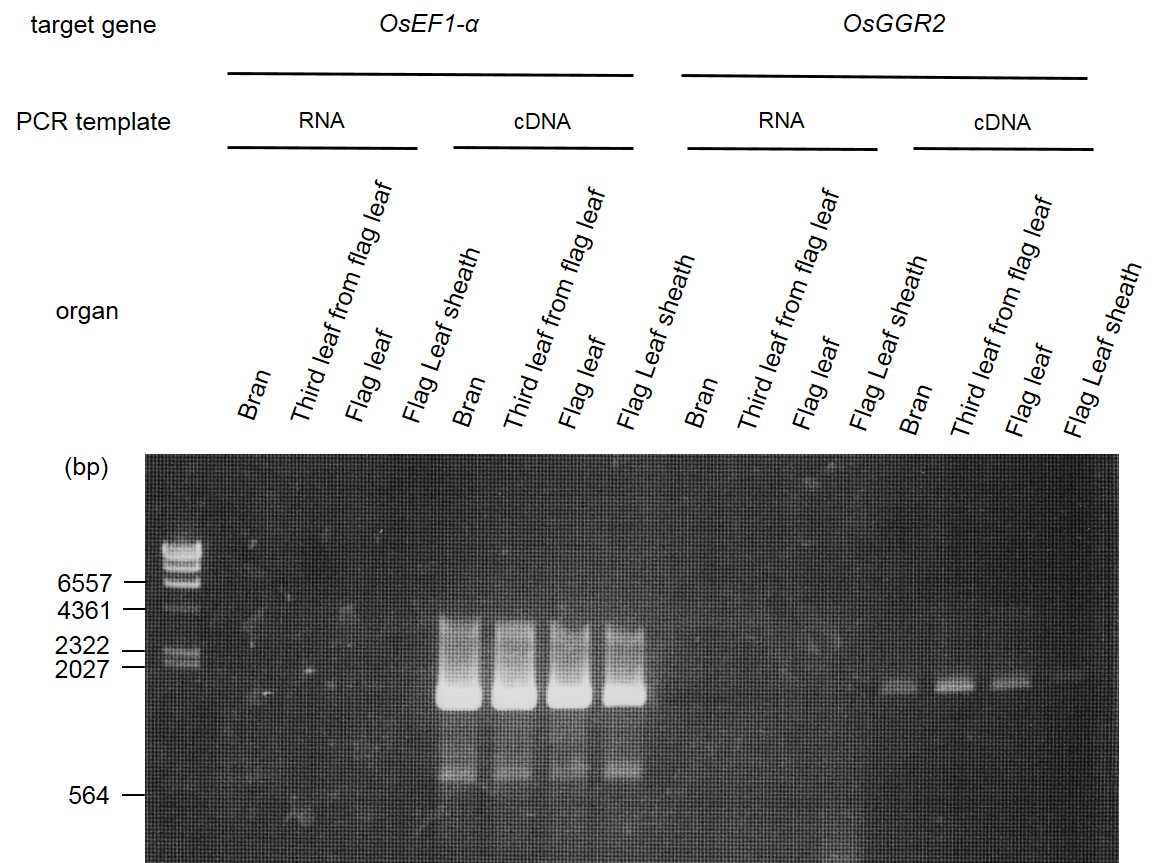
a**

**
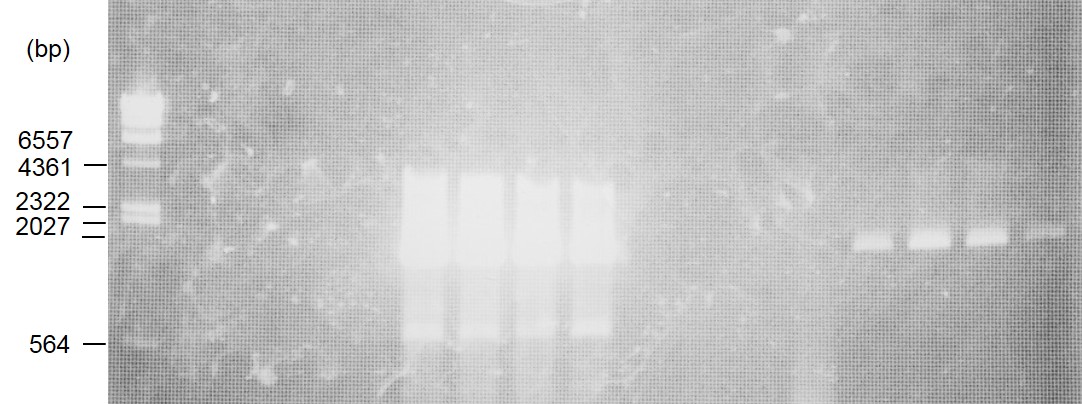
 b**

**Supplementary Figure S3. Full-length Gel from the article of Fig. 4D.** Fig. S3b is high-exposure gel of Fig.S3a.

**
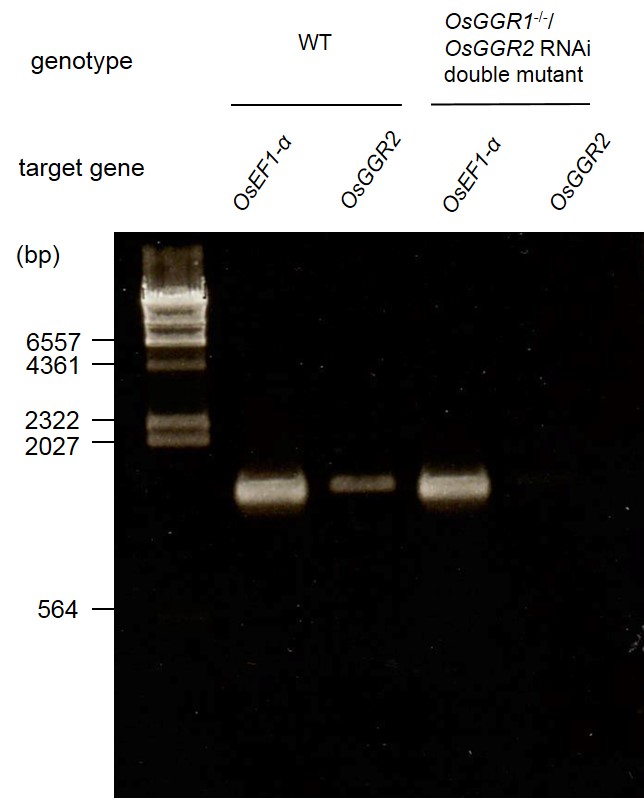
**

**Supplementary Figure S4. Full-length Gel from the article of Fig. 5B.**

**Table S1: List of primer pairs sequences for PCR.**

**Primers for genotyping analysis of *OsGGR1 Tos17* mutant**

**Forward primer 5'- GGCGTGTGAACAACTAGTGC -3'**

**Reverse primer 1 5'- GTACGTGACAAAGTGCTCCG -3'**

**Reverse primer 2 5'- TCCACCTTGAGTTTGAAGGG -3'**

**Primers for gene expression analysis**

***OsGGR1***

**Forward primer 5'- CACCATGACCTCGCTGTCGTCCTCC -3'**

**Reverse primer 5'- CCGGAGCACTTTGTCACGTACC -3'**

***OsGGR2***

**Forward primer 5'- CACCCAATCTTCCACTCGCATCTCATC -3'**

**Reverse primer 5'- AGAGCAGTACACACATCTTCAATG -3'**

***OsEF1-α***

**Forward primer 1 5'- ATGGGTAAGGAGAAGACGCACATC -3'**

**Reverse primer 1 5'- GAGACTTCCTTCACGATTTCATC -3'**

**Forward primer 2 5'- CAGCTACCTGCTTAATCAACC -3'**

**Reverse primer 2 5'- TCATTTCTTCTTGGCGGCAGCC -3'**

**Primers for *OsGGR2* cloning**

**Forward primer 5'- CACCAATTTGAGCTGTGGCGCCATCTC -3'**

**Reverse primer 5'- GGGTTGCGCTCGAGGAGGAAC -3'**

**Primers for *OsGGR2* RNAi**

**Forward primer 5'- CACCAATTTGAGCTGTGGCGCCATCTC -3'**

**Reverse primer 5'- GCGAACTCGTCGAGCATGCAG -3'**
